# Improvement in fruit yield and tolerance to salinity of tomato plants fertigated with micronutrient amounts of iodine

**DOI:** 10.1038/s41598-022-18301-w

**Published:** 2022-08-29

**Authors:** Claudia Kiferle, Silvia Gonzali, Sara Beltrami, Marco Martinelli, Katja Hora, Harmen Tjalling Holwerda, Pierdomenico Perata

**Affiliations:** 1 PlantLab, Center of Plant Sciences, Scuola Superiore Sant’Anna, Pisa, Italy; 2 SQM International N.V., 2030 Antwerpen, Belgium

**Keywords:** Physiology, Plant sciences

## Abstract

Iodine is an essential micronutrient for humans, but its role in plant physiology was debated for nearly a century. Recently its functional involvement in plant nutrition and stress-protection collected the first experimental evidence. This study wanted to examine in depth the involvement of iodine in tomato plant nutrition, also evaluating its potential on salt stress tolerance. To this end, iodine was administered at dosages effective for micronutrients to plants grown in different experimental systems (growth chamber and greenhouse), alone or in presence of a mild-moderate NaCl-salinity stress. Plant vegetative fitness, fruit yield and quality, biochemical parameters and transcriptional activity of selected stress-responsive genes were evaluated. In unstressed plants, iodine increased plant growth and fruit yield, as well as some fruit qualitative parameters. In presence of salt stress, iodine mitigated some of the negative effects observed, according to the iodine/NaCl concentrations used. Some fruit parameters and the expressions of the stress marker genes analyzed were affected by the treatments, explaining, at least in part, the increased plant tolerance to the salinity. This study thus reconfirms the functional involvement of iodine in plant nutrition and offers evidence towards the use of minute amounts of it as a beneficial nutrient for crop production.

## Introduction

Nowadays, 632 million hectares of agricultural land, corresponding to one fifth of the total world’s cultivable soil, are classified as salt-affected^1^. Salinity is considered one of the most important abiotic stresses threatening agricultural productivity due to the detrimental effects on plant production and yield^2^. Excess salt in the soil reduces the ability of the plant to absorb water, leading to osmotic stress and ion toxicity for the excessive accumulation of Cl^−^ and Na^+^^3,4^. These in turn lead to a series of secondary effects such as nutrient imbalance, oxidative stress, and inhibition of photosynthesis, dampening plant growth and production^4^. Plant adaptation to saline conditions includes activation of different biochemical and physiological strategies aimed at restoring ion and water homeostasis^5^.

In the last decades several components of salt tolerance have been characterized in plants, providing the basis for the development of more tolerant varieties by either conventional breeding or genetic modifications^6^. Among horticultural crops, tomato (*Solanum lycopersicum* L.) is one of the most important model species, especially useful to study salt tolerance due to its well-known genetics and convenient transforming techniques^7,8^. The physiology of tomato in saline and not saline conditions has been largely characterized. Tomato is considered as “moderately tolerant” to salinity due to its ability to regulate water and ionic homeostasis at moderate levels of salinity in the root zone^9^. Nevertheless, exposure to high salt concentrations is known to cause negative effects in most of its cultivars in terms of seed germination, inhibition of growth and reduction of fruit productivity^8^. Leaf growth inhibition has also been observed in plants exposed to excess salinity in the root zone and has been attributed to reduced cellular turgor, diminished photosynthetic activity and activation of metabolic signaling between stress perception and adaptation^10–12^.

The correct fertilization of crops, and in particular the exogenous application of mineral micronutrients, has emerged as a promising approach to partially mitigate the adverse effects of different abiotic stresses, including salinity^13^. An increasing number of studies include results showing that exogenous application of iodine—in dosages corresponding to a micronutrient application—beneficially affects redox metabolism^14,15^ and stimulates non-enzymatic and enzymatic antioxidant synthesis, thus increasing tolerance to various adverse conditions, including salinity^3,16,17^. However, the potential of iodine to induce tolerance to salinity stress in the context of plant nutrition is still poorly reported in literature. In a trial with lettuce, the exogenous application of iodine in the form of KIO_3_ has been found to increase the activity of the main detoxifying enzymes of Reactive Oxygen Species (ROS), such as Superoxide Dismutase (SOD), Ascorbate Peroxidase (APX), and Catalase (CAT), thus increasing the plant’s capacity to tolerate severe salinity stress^3^. Similar results have recently been found in tomato^18^, where the foliar application of iodine was found to enhance the antioxidant capacity of the seedlings and to increase their tolerance during salt exposure.

Very recently, the nutritional role of iodine has been demonstrated, as this element can be covalently bound to at least 82 different proteins in *Arabidopsis thaliana* leaves and roots^15^. The occurrence of protein iodination was additionally demonstrated in phylogenetically distant species, such as tomato, lettuce, wheat, and corn. In Arabidopsis, the presence of iodine in micromolar concentrations in the nutrient solution also resulted in increased plant biomass accumulation and timely flowering, compared to iodine-deficient controls^15^.

In the present study, the role of iodine in tomato plant nutrition was investigated, focusing on its potential to improve salinity stress tolerance. At this aim, a first small-scale experiment was performed to evaluate the effect of iodine on the vegetative fitness and fruit production of the tomato model cultivar Micro-Tom, exposed to different salinity levels in a growth chamber. Secondly, a commercial-scale experiment was performed in a greenhouse with a conventional hybrid tomato variety. The effect of iodine, supplied alone at different levels or in combination with diverse salt stress conditions, was verified in terms of plant vegetative fitness, leaf proline content, fruit yield and quality, iodine content and transcriptional activity of selected stress-responsive genes, including those involved in the antioxidant metabolism.

## Results

### Growth chamber experiment

The goal of the experiment was to evaluate the effect of soil iodine treatments, provided as 50 and 100 μM KIO_3_, on the main tomato vegetative parameters and fruit production, analyzing, as well, its possible protective role against 25, 50 or 150 mM NaCl salt stress. Iodine and NaCl were simultaneously added to the basal fertilizing solution starting from two weeks following seed germination, until fruit collection (3 treatments/week; 10 weeks, for a total number of 30 treatments).

**Figure 1:**
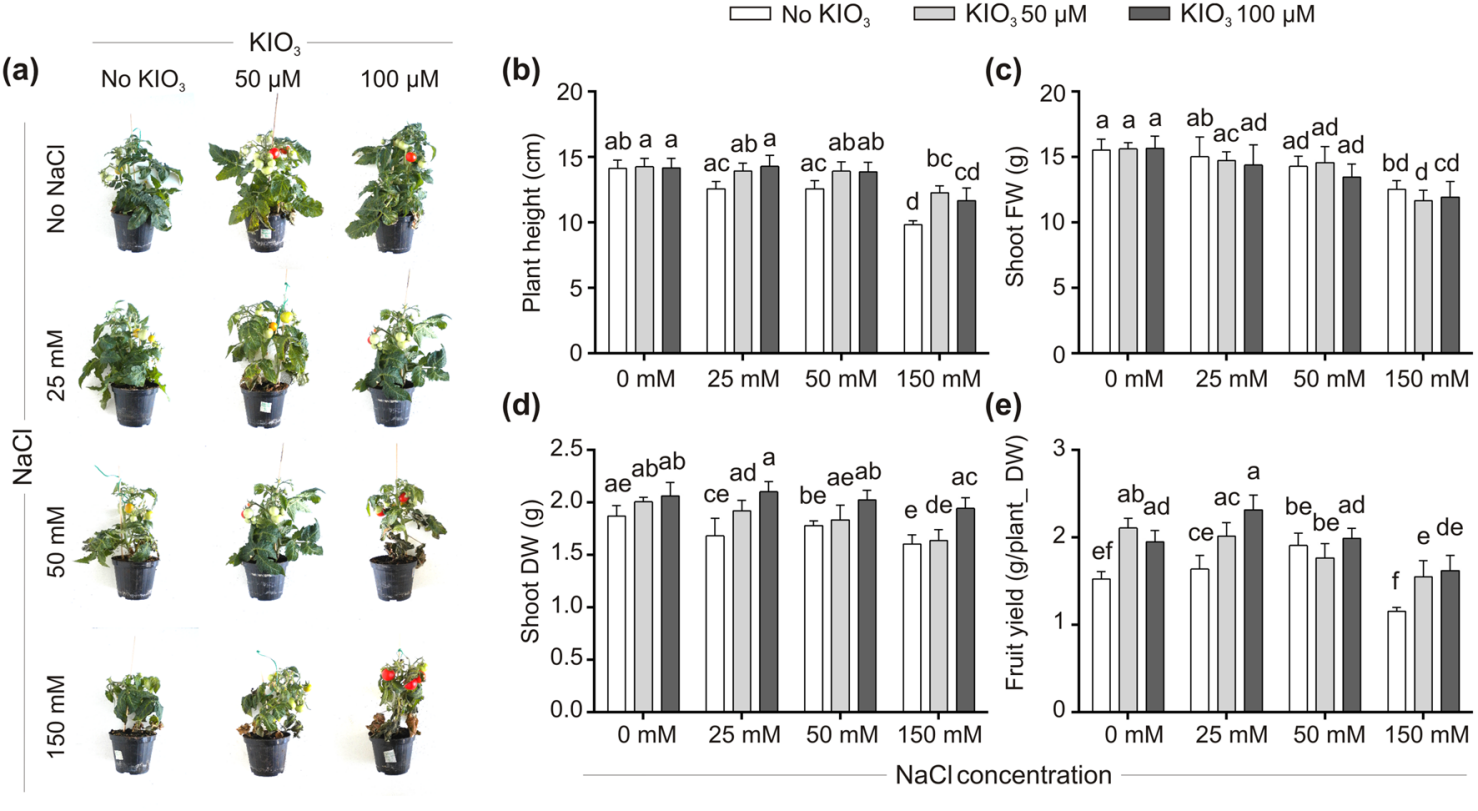
Growth chamber experiment: impact of iodine on plant vegetative fitness and its adaptation to salt stress. Lateral view of plants after 10 weeks from the onset of NaCl and/or KIO_3_ treatments (**a**). Plant height (**b**), shoot FW (**c**) and DW (**d**) and plant yield (**e**). Each bar is the mean (± SE) of 12 replicates, each consisting of one individual plant. When data followed a Normal distribution and there was homogeneity of variances, they were subjected to one-way ANOVA and values indicated by different letters significantly differ from each other (LSD post hoc test, P ≤ 0.05). When one of this two prerequisites was violated, a Kruskal–Wallis test was performed and significant differences within medians were determined by Box-and-Whisker Plot (median notch option, P ≤ 0.05) and indicated by different letters.

In the absence of NaCl, iodine treatments, although not influencing plant height (Fig. 1b) or shoot fresh weight (FW) (Fig. 1c), positively affected the shoot biomass production (Fig. 1d). Moreover, they had a remarkable effect on fruit yield, which was approximately 39.5% and 29% higher, compared to the control, in plants supplied with 50 and 100 μM KIO_3_, respectively (Fig. 1e).

The harmful effect of salinity was particularly evident in 150 mM NaCl-treated plants (Fig. 1a), as a considerable decrease in plant height (Fig. 1b), shoot FW and dry weight (DW) (Fig. 1c,d) and fruit production (Fig. 1e) was observed compared to the unstressed plants. Iodine, when added together with NaCl, promoted plant vegetative fitness and fruit production, strongly mitigating the negative effects induced by the higher NaCl dose applied (Fig. 1a,b,d,e). Apart from the shoot FW, all the vegetative and production parameters indeed did not differ from those of the unstressed plants. In addition, compared with the iodine-unenriched plants, 100 μM KIO_3_ significantly increased the fruit production of 25 and 150 mM NaCl-treated plants by 41.5% and 42% respectively (Fig. 1e), also increasing by 25% and 21% their shoot DW (Fig. 1d).

### Greenhouse experiment

**Figure 2:**
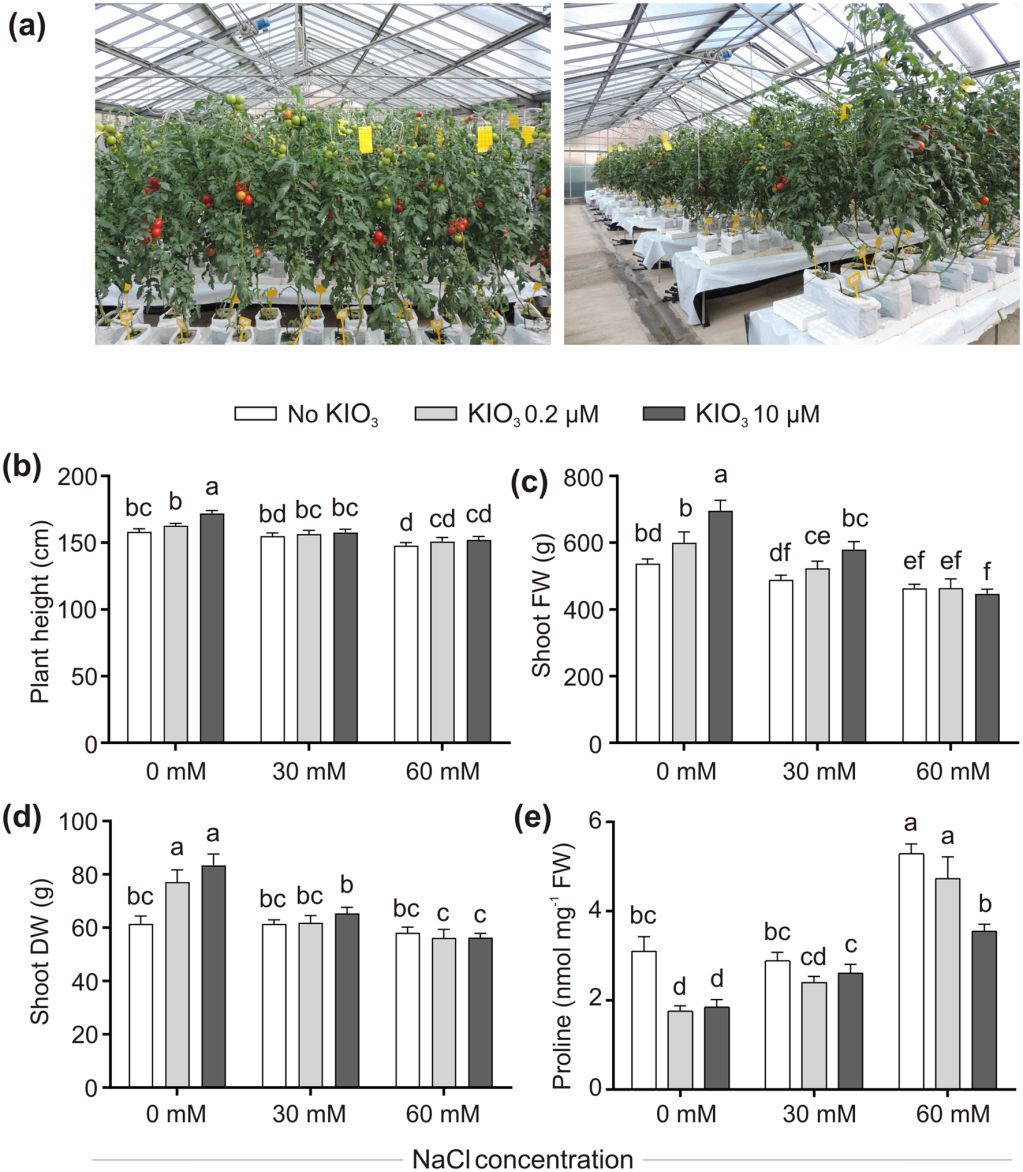
Greenhouse experiment: impact of iodine on plant vegetative fitness and its adaptation to salt stress. Overview of the hydroponically grown plants, under greenhouse conditions (**a**). Plant height (**b**), shoot FW (**c**) and DW (**d**) and leaf proline content (**e**). Each bar is the mean (± SE) of 13 replicates, each consisting of one individual plant (**b**, **c**, **d**), or three replicates (**e**), each consisting in a pool of the same-age terminal leaflets harvested from different plants. When data followed a Normal distribution and there was homogeneity of variances, they were subjected to one-way ANOVA and values indicated by different letters significantly differ from each other (LSD post hoc test, P ≤ 0.05). When one of this two prerequisites was violated, a Kruskal–Wallis test was performed and significant differences within medians were determined by Box-and-Whisker Plot (median notch option, P ≤ 0.05) and indicated by different letters.

An experimental set-up closer to a normal commercial cultivation of tomato was used, by growing plants in a hydroponic system under greenhouse conditions (Fig. 2a).

In the previous experiment, the positive effects of iodine were already evident at the lowest concentration applied (50 μM KIO_3_). For this reason, also considering that in the greenhouse trial both the iodine and the NaCl salts would have been directly added to the daily supplied nutrient solution of the hydroponic system, which would facilitate a better uptake of all ions by the plants, the concentrations of both the salts administered were strongly reduced. Iodine, in the form of KIO_3_ and at the doses of 0.2 and 10 μM, was thus added to the fertigation solution alone or in combination with 30 or 60 mM NaCl, corresponding to a mild or a moderate salt stress, respectively. Iodine and/or NaCl treatments were performed starting from 3 weeks following the transplant, until the end of the trial (complete ripening of fruits developed on the 5th truss).

**Figure 3:**
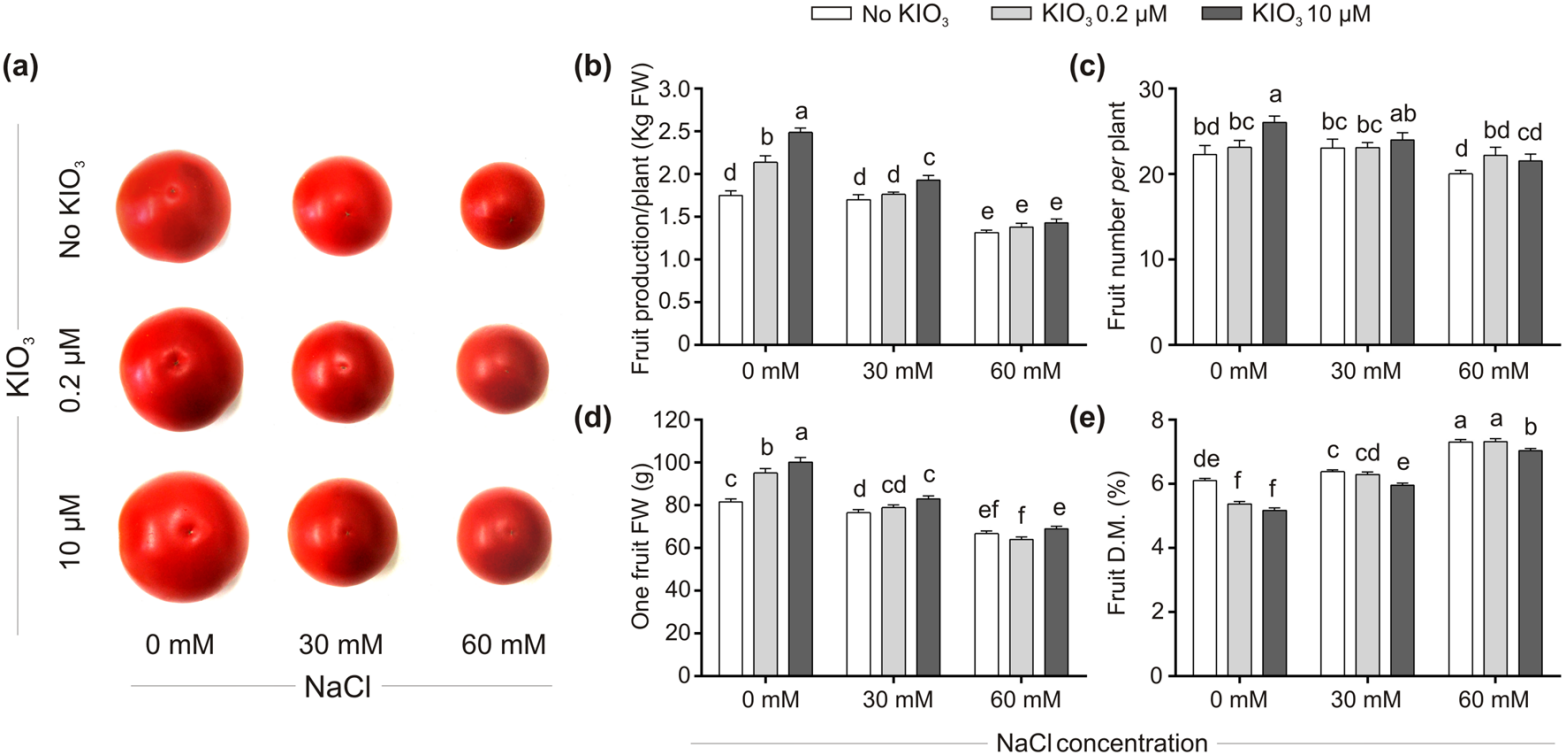
Impact of iodine on fruit yield in the absence/presence of salt stress. Representative fruits at harvest (**a**). Fruit yield (**b**) and number of produced fruits/plant (**c**), determined by collecting all the fruits produced during the growing cycle on each truss. Each bar is the mean (± SE) of 9 replicates, each consisting of the averaged values of fruits collected from each truss, shared on different benches. Individual fruit FW (**d**) and dry matter percentage (D.M.%; **e**). Each bar is the mean (± SE) of 135 replicates (27 fruits·5 trusses), each consisting of one individual fruit. When data followed a Normal distribution and there was homogeneity of variances, they were subjected to one-way ANOVA and values indicated by different letters significantly differ from each other (LSD post hoc test, P ≤ 0.05). When one of this two prerequisites was violated, a Kruskal–Wallis test was performed and significant differences within medians were determined by Box-and-Whisker Plot (median notch option, P ≤ 0.05) and indicated by different letters.

**Figure 4:**
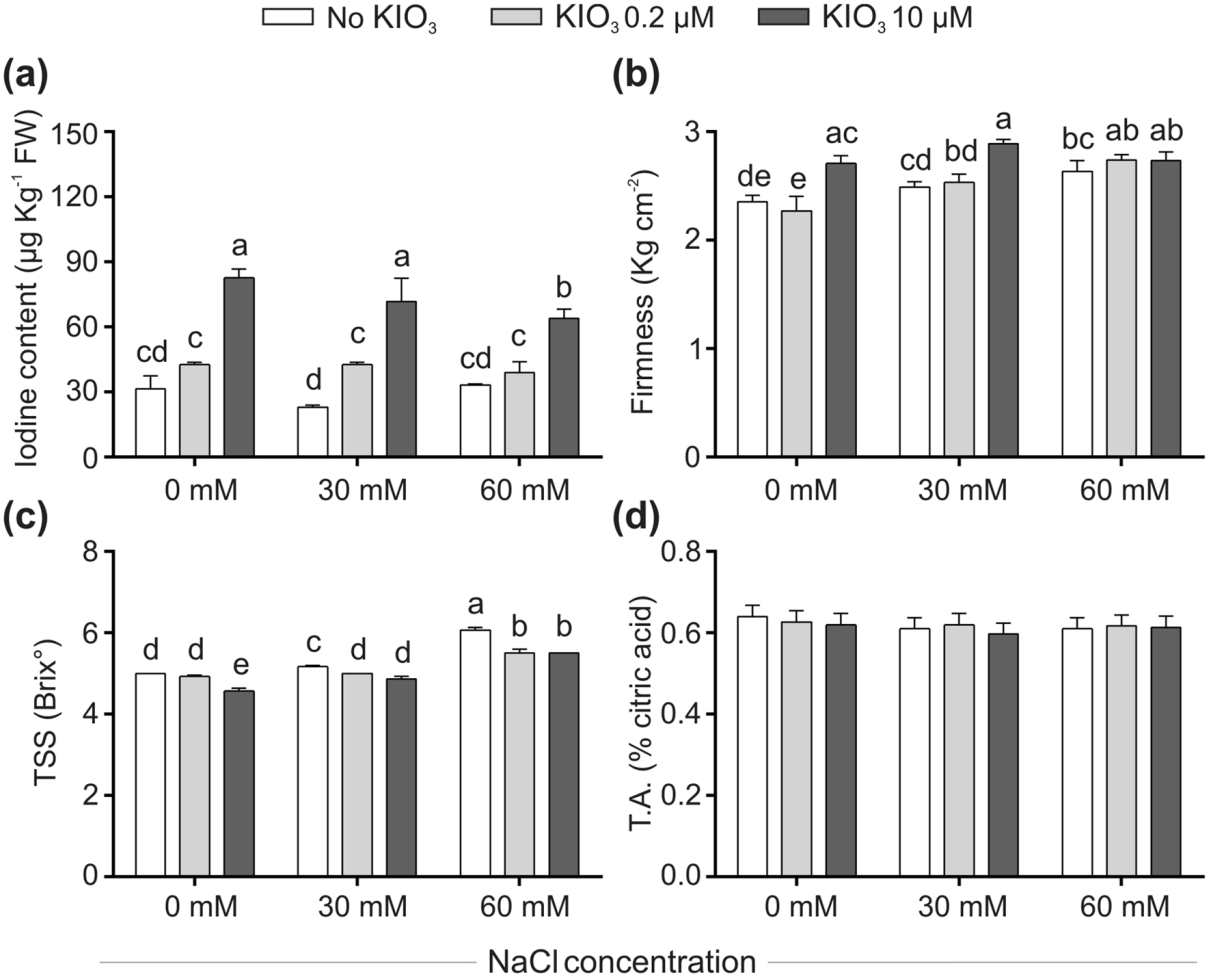
Impact of iodine on fruit quality in the absence/presence of salt stress. Iodine content (**a**), firmness (**b**), total soluble solids (TSS; measured as °Brix; **c**) and titratable acidity (T.A.; **d**) are shown. All the fruits produced in the 2nd position of the 3rd truss cluster (n = 27) were collected and used for qualitative determinations. In the fruit firmness graph, each bar is the mean (± SE) of 12 replicates, each consisting of one individual fruit/plant. In the other graphs each bar is the mean (± SE) of 3 replicates, each consisting of a sub-sample produced through homogenization of the remaining collected material (27–12 = 15 fruits). When data followed a Normal distribution and there was homogeneity of variances, they were subjected to one-way ANOVA and values indicated by different letters significantly differ from each other (LSD post hoc test, P ≤ 0.05). When one of this two prerequisites was violated, a Kruskal–Wallis test was performed and significant differences within medians were determined by Box-and-Whisker Plot (median notch option, P ≤ 0.05) and indicated by different letters.

**Figure 5:**
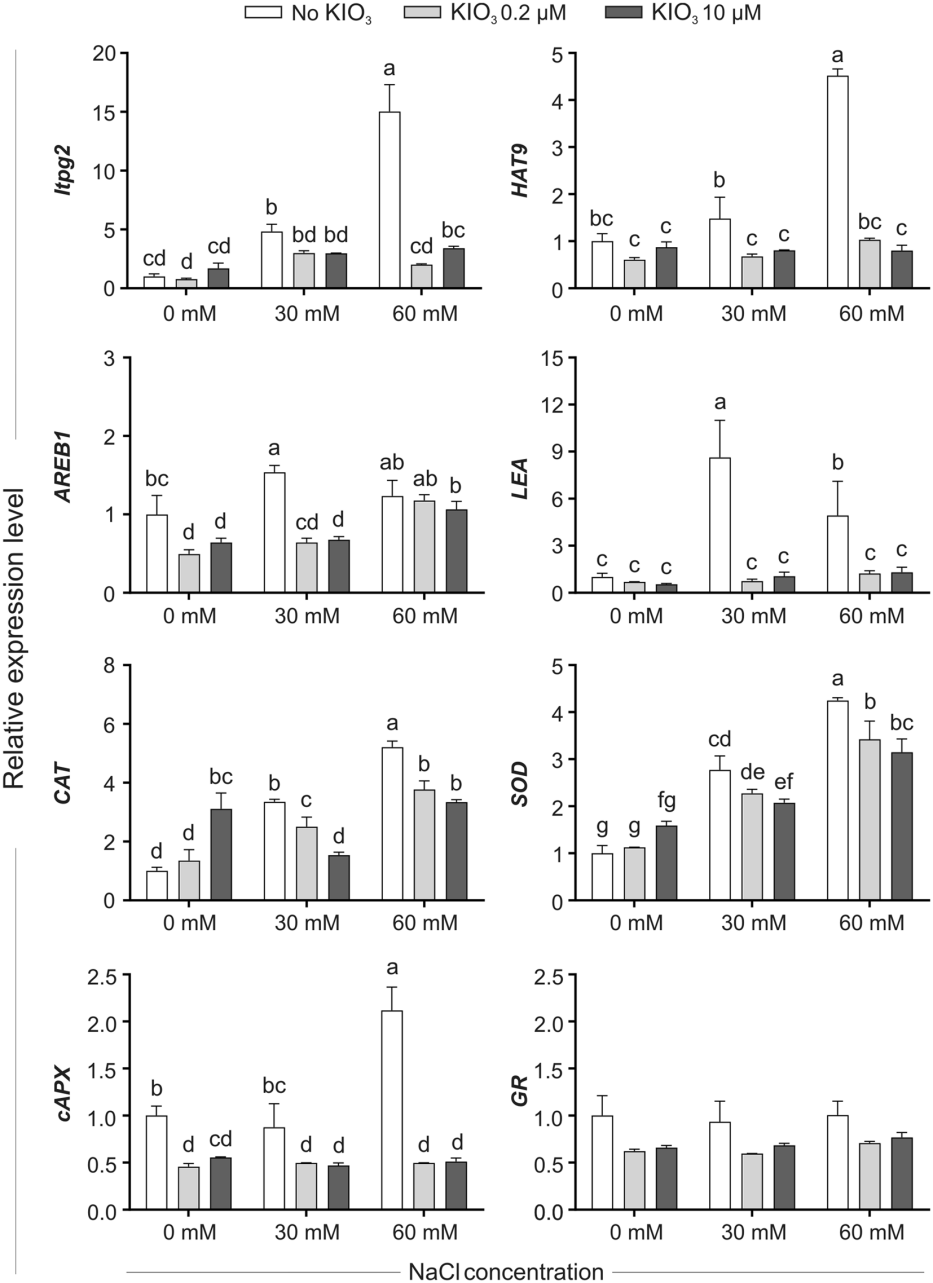
Expression level of the selected stress-related genes in leaf samples collected after 72 h from the beginning of the KIO_3_ and/or NaCl administration. qPCR data are means (± SE) of three biological replicates, each consisting of a pool of leaf samples, and are expressed as relative units, setting to one the mean value of unstressed plants, not treated with iodine. When data followed a Normal distribution and there was homogeneity of variances, they were subjected to one-way ANOVA and values indicated by different letters significantly differ from each other (LSD post hoc test, P ≤ 0.05). When one of this two prerequisites was violated, a Kruskal–Wallis test was performed and significant differences within medians were determined by Box-and-Whisker Plot (median notch option, P ≤ 0.05) and indicated by different letters. *lptg* gene encoding a non-specific lipid transfer protein, *HAT9* gene encoding a homeobox-leucine zipper protein, *AREB1* gene encoding a bZIP transcription factor, *LEA* gene encoding a late embryogenesis abundant protein, *CAT* Catalase, *SOD* Superoxide Dismutase, *cAPX* cytosolic Ascorbate Peroxidase, *GR* Glutathione Reductase.

**Figure 6:**
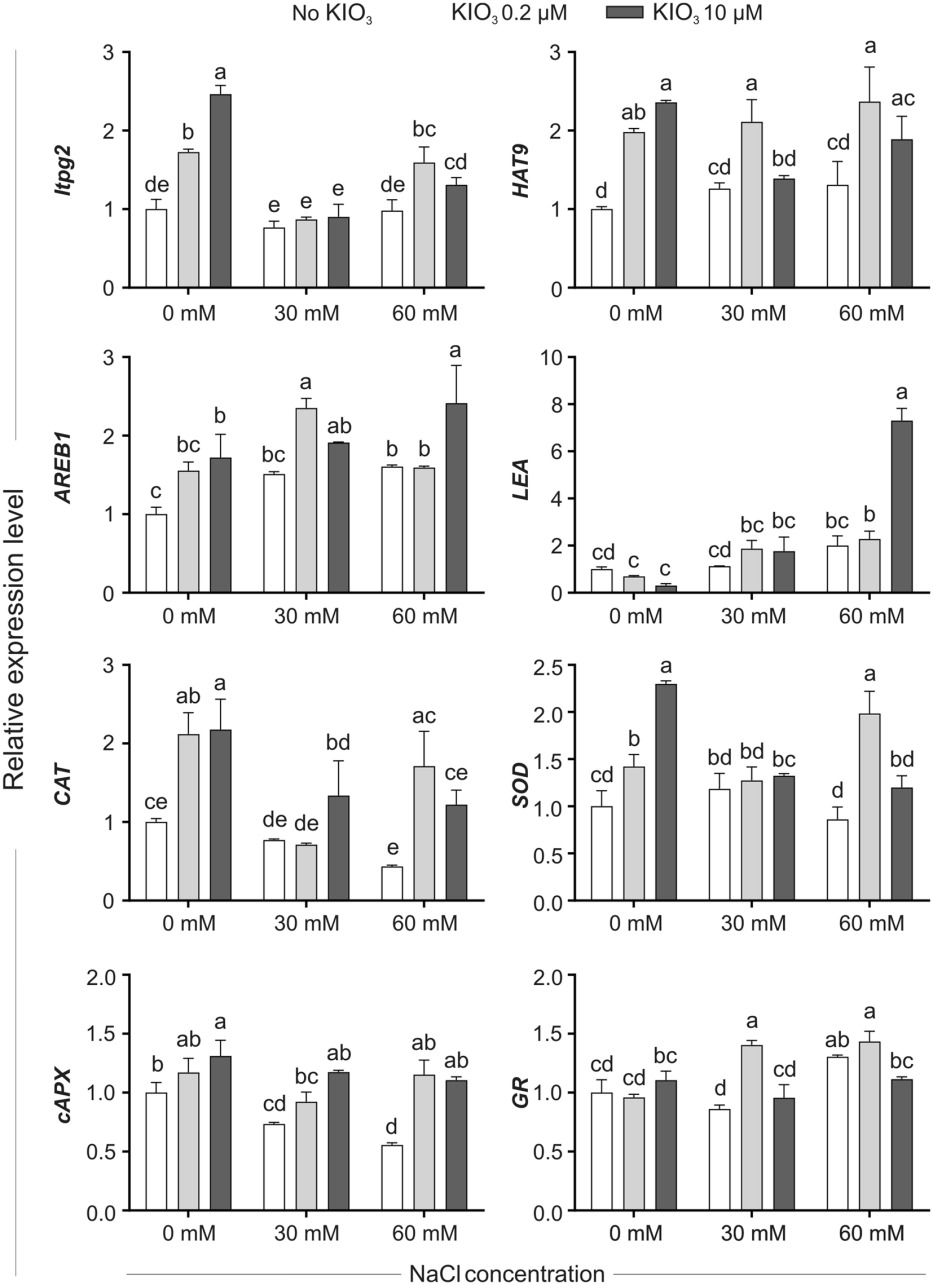
Expression level of the selected stress-related genes in leaf samples collected after 8 weeks from the beginning of the KIO_3_ and/or NaCl administration. qPCR data are means (± SE) of three biological replicates, each consisting of a pool of leaf samples, and are expressed as relative units, setting to one the mean value of unstressed plants, not treated with iodine. When data followed a Normal distribution and there was homogeneity of variances, they were subjected to one-way ANOVA and values indicated by different letters significantly differ from each other (LSD post hoc test, P ≤ 0.05). When one of this two prerequisites was violated, a Kruskal–Wallis test was performed and significant differences within medians were determined by Box-and-Whisker Plot (median notch option, P ≤ 0.05) and indicated by different letters. *lptg* gene encoding a non-specific lipid transfer proten, *HAT9* gene encoding a homeobox-leucine zipper protein, *AREB1* gene encoding a bZIP transcription factor, *LEA* gene encoding a late embryogenesis abundant protein, *CAT* Catalase, *SOD* Superoxide Dismutase, *cAPX* cytosolic Ascorbate Peroxidase, *GR* Glutathione Reductase.

The effects of the treatments were monitored in terms of the main plant vegetative parameters and leaf proline content (Fig. 2), fruit yield (Fig. 3) and quality (Fig. 4), and on the expression of selected stress-responsive genes (Figs. 5, 6).

#### Effects of iodine on plant vegetative parameters, fruit yield and tolerance to salt stress—greenhouse experiment

No phytotoxicity symptoms were observed on plants during the whole growing cycle, irrespective of the NaCl or iodine treatments performed (Fig. 2a). In the absence of NaCl, iodine impressively improved the vegetative growth of tomato plants (Fig. 2b,c). This was particularly evident in 10 μM KIO_3_-treated plants, even if the addition of iodine at 0.2 µM could also exert positive, although not always statistically significant, effects on the detected parameters. In detail, 0.2 µM and 10 µM KIO_3_ increased the plant height by 2.9% and 8.8% (Fig. 2b), the shoot FW by 11.5% and 29.4% (Fig. 2c) and its DW by 25.5% and 35.6% (Fig. 2d), respectively, when compared with the iodine-untreated ones. The shoot water content was not affected by iodine or NaCl, as demonstrated by the plant dry matter percentage, whose values remained unchanged in all the conditions tested (Supplementary Fig. S1). The lower salt stress applied (30 mM NaCl) scarcely affected the plant vegetative fitness (Fig. 2b–d), which was instead promoted by the simultaneous addition of KIO_3_: the shoot FW was indeed increased by 16.7% and 29.2%, respectively, from addition of iodine at 0.2 µM and 10 µM, when compared with the iodine-untreated ones (Fig. 2c). Iodine could partially prevent the negative effect on plant height induced by the highest NaCl concentration tested (60 mM NaCl) (Fig. 2b), not mitigating the concomitant reduction of plant shoot FW (Fig. 2c).

In addition, iodine strongly influenced the leaf proline content (Fig. 2e): in the absence of salt stress, 0.2 µM and 10 µM KIO_3_, reduced more than 1.5-fold its accumulation in comparison with the iodine-untreated samples. A similar behavior could be observed in the presence of salt stress, especially in plants treated with 10 µM KIO_3_ and exposed to 60 mM NaCl (Fig. 2e).

In the absence of salt stress, the fruit production was astonishingly improved by both the iodine treatments (Fig. 3a,b): the application of iodine at 0.2 µM and 10 µM increased fruit yield by approximately 22% and 42% compared to their control, respectively. The increased productivity was due to the combination of the promoting effect of iodine at 0.2 µM and 10 µM on the number of produced fruits (more than 3.7% and 17%, respectively, of those from the iodine-untreated plants; Fig. 3c) and on their FW (more than 17% and 23%, respectively, of iodine-untreated plants; Fig. 3d). The fruit dry matter content was significantly reduced by iodine (approximately by 11.5% and 15%, in 0.2 µM and 10 µM KIO_3_-treated plants in comparison with iodine-untreated plants, respectively; Fig. 3e).

Iodine alleviated the detrimental effect induced by the mild salinity stress (30 mM NaCl). The highest iodine concentration tested (10 µM KIO_3_), significantly improved the fruit FW (more than 8.2% of iodine-untreated plants; Fig. 3d) and the final plant yield (more than 13.5% of iodine-untreated plants; Fig. 3b). Fruits of 10 µM KIO_3_-treated plants also accumulated more water, as suggested by the decrease in their dry matter percentage (Fig. 3e). When exposed to moderate salt stress (60 mM NaCl), iodine-treated plants tended to produce more and bigger fruits than the controls (Fig. 3a,c), even if this positive effect was not enough to significantly increase the fruit yield.

#### Effects of iodine on fruit quality, in the presence/absence of salt stress—greenhouse experiment

The effect of iodine on fruit quality was evaluated by determining their total soluble solids (TSS) content, titratable acidity, firmness (Fig. 4), carbohydrate (glucose, fructose and sucrose) and protein content (Supplementary Fig. S2), and peel colour (Supplementary Fig. S3). KIO_3_ treatments increased the fruit iodine concentration in a dose–response manner, ranging from 29 μg/kg FW (control plants) to 40 and 80 μg/kg FW in 0.2 µM and 10 µM iodine-treated plants, respectively (average values of controls and salt-treated plants at different KIO_3_ concentrations; Fig. 4a). The concomitant presence of NaCl in the nutrient solution did not interfere with iodine uptake and/or transport to fruits in 0.2 µM iodine-treated plants, whereas a slight negative interference of 60 mM NaCl was observed on this trait at the higher iodine dose applied (10 µM) (Fig. 4a).

Fruit firmness tended to increase with increasing salinity levels (Fig. 4b). 10 µM KIO_3_ positively affected this parameter in unstressed plants and in those exposed to mild salt stress, whereas 0.2 µM KIO_3_ did not result in any significant alteration of this trait at all NaCl concentrations tested.

In the absence of iodine, salinity increased the fruit TSS in a dose dependent manner (Fig. 4c). On the contrary, a negative impact of iodine on TSS was observed, at all salinity levels. The same behaviour could be observed on glucose content (Supplementary Fig. S2a), whereas the concentrations of fructose and sucrose were significantly affected neither by salinity nor by iodine treatments (Supplementary Fig. S2b,c), as well as the proteins concentration (Supplementary Fig. S2d). In a similar way, fruit titratable acidity (Fig. 4d) and colour (Supplementary Fig. S3) were affected neither by iodine nor by salt stress.

#### Effects of iodine on gene expression, in the presence/absence of salt stress—greenhouse experiment

To examine more in depth the possible role of iodine in tomato plants, the effects on plant vegetative fitness and fruit production were correlated with physiological processes that may be affected by iodine. For this purpose, the transcriptional response to iodine in presence or absence of salt stress was determined on a selection of genes involved in the plant osmotic and antioxidant response. The first group included some well-characterized stress marker genes, mainly involved in ABA-related responses affecting dehydration and osmoregulation processes (*AREB1*^19^, *lptg2*^20^, *LEA*^21^, *HAT9*^21^). The second group included genes which can be involved in the oxidative system of the plant: *CAT*, *SOD*, *cAPX*, and *GR*^22^. We decided to follow the transcriptional response of these two classes of genes as a number of studies focuses on the link between salt stress and antioxidant systems in combination with ABA biosynthesis and signalling^23^.

Early and late transcriptional events induced by salt and/or iodine administrations were characterized by analysing leaf samples collected 72 h and 8 weeks after the onset of the treatments, respectively.

In the absence of iodine, most of the stress marker genes analysed 72 h after the onset of the treatments appeared to be more expressed in NaCl-treated plants, compared to unstressed ones (Fig. 5). Notably, for some of these genes (*ltpg2*, *HAT9*, *CAT*, c*APX* and *SOD*), the transcriptional induction was correlated with the level of the salt supplied, being higher in the presence of 60 mM than of 30 mM NaCl (Fig. 5). Iodine (at both the concentrations supplied) diminished the NaCl activation of the stress marker genes *ltpg2*, *HAT9*, *AREB1*, *LEA*, *CAT*, *SOD* and *cAPX*. On the contrary, the transcriptional response of unstressed plants was not particularly influenced by iodine treatments: the expression level of most of the genes was indeed similar in iodine-treated and not treated plants, or at most only slightly affected in the first ones (as in the case of *AREB1*, *CAT*, and *cAPX*) (Fig. 5).

At 8 weeks after the onset of the treatments, the expression level of most of the selected genes in not-iodine-treated plants was comparable in control and NaCl-treated plants (Fig. 6). On the contrary, a clear activation of *ltpg2*, *HAT9*, *AREB1*, *CAT*, *SOD* and c*APX* was observed in plants not submitted to salinity and treated with increasing levels of iodine (Fig. 6). Furthermore, almost all the genes which were down-regulated by iodine in NaCl-treated plants during the early sampling (Fig. 5) were slightly activated at 8 weeks after the onset of the treatments, even if at different extent, depending on the iodine/NaCl combination used and the specific gene analyzed (Fig. 6). The expression of these genes increased upon iodine + salt treatment, and was in general higher than observed under salt or iodine treatment alone.

## Discussion

The recent findings on the effects of iodine in *Arabidopsis thaliana*^15^ called for a reassessment of the role of this element as a plant nutrient in a commercially relevant crop such as tomato^24^. Also, there was a need to confirm that the low micromolar doses that were effectively used in Arabidopsis would still be effective in tomato in a commercial production setting. Furthermore, the potential role of iodine in resilience to abiotic stresses as found in Arabidopsis^15^ and supported by a series of scattered but consistent bibliographic indications^3,18,25^ had to be confirmed.

The first result of the present study was that in both the experimental set-ups (growth chamber and greenhouse) carried out we observed, more than expected, that iodine treatments induced an impressive increase in the total fruit yield (Figs. 1e, 3b), which is the most relevant commercial trait for tomato, also associated with a beneficial effect on plant growth (Figs. 1c, 2c). The higher fruit yield was due to a combination of an increased number of fruits produced *per* plant and of an increased average fruit weight (Fig. 3b–d).

This result was not completely unexpected, since positive effects of 1–10 µM iodine on plant vegetative fitness and yield were already reported in tomato and other crops, such as spinach^26^, lettuce^16^, strawberry^27^, barley^28^, and wheat^29^. In accordance with our data, Borst Pauwels^28^ observed a stimulating effect of low doses of iodine on longitudinal and radial growth of tomato plants and fruit dry matter yield. In addition, Lehr et al.^30^ demonstrated in a three-year experiment that 12 µM KI increased tomato yield in a 10–76% range in comparison with control plants, which prompted the first assumption of an essential role of iodine in tomato plant nutrition. A positive effect of the organo-iodine compound 5-iodosalycilic acid on the leaves biomass of young tomato plants has also been recently described at doses ranging from 5 to 25 μM^31^. Nevertheless, contrasting results were also reported. Other studies on tomato indicated, for example, that the addition of 5–50 μM iodine, supplied as KI, KIO_3_ or organic iodine forms, was not detrimental for plant growth, but did not result in an increase in terms of shoot biomass and fruit production^32–34^. Adverse effects of iodine have been even observed on plants, and associated with severe phytotoxicity symptoms, if supplied at high concentrations and/or in the I^−^ form^17,35^. A possible explanation for this seemingly contradictory evidence is the very common availability of iodine in water, soil, and atmosphere^36^, from which plants can freely absorb, through roots and leaves, those minute amounts of the element which are probably sufficient for their life and development. Consequently, the experimental set-ups used to carry out the different studies may not be comparable, and the lack of a precise knowledge of the natural iodine availability in the environment where plants are grown makes it very difficult to understand which part of the exogenously applied iodine is truly taken up by plants and responsible for the described effects. Moreover, it is still not clear whether the mechanisms of iodine volatilization as methyl iodide are widespread in higher plants and used as effective means to modulate iodine concentrations in plant tissues or only serve to eliminate excess iodine to avoid its possible phytotoxicity^35,37^.

In our trials, we used Milli-Q water in the growth chamber experiment and measured the iodine concentrations in the tap water used in the greenhouse, to be sure that the amount of iodine received in control conditions was negligible compared to the quantity added to the nutrient solution. Despite these precautions, low amounts of iodine were detected in fruits collected from iodine-untreated plants (Fig. 4a). These may be attributed to the traces of iodine (0.03 µM) present in the tap water used to prepare the nutrient solutions and indicate a high ability of tomato plants to mobilize iodine to fruits even when present in the environment in subtle amounts. Moreover, considering the randomized distribution of treatments in the greenhouse (Supplementary Fig. S5), we cannot exclude the possibility that methyl iodide emissions^37^ from iodine-treated plants constituted a source of “contamination” for the iodine-untreated ones. In any case, in our experiments the possible external sources of iodine were contained as much as possible and the results we obtained can be mostly ascribed to the controlled iodine treatments performed.

Besides the quantitative effects on plant growth and yield, iodine administration also stimulated qualitative changes of some fruit parameters. Fruit organoleptic quality is strongly affected by the content of TSS and organic acids, as they influence sweetness, sourness and flavour intensity^38^. Tomato fruit TSS reflects the dry matter content and is inversely proportional to fruit size and moisture level^39^. In our study, iodine treatments slightly reduced the fruit TSS (Fig. 4c), as observed in previous studies^40,41^, as well as its glucose content (Supplementary Fig. S2a), probably as a consequence of the increase in fruit water accumulation (Fig. 3e). In spite of that, the fruit protein content, which is one of the major class of nutrients, was not affected (Supplementary Fig. S2d). Iodine did not alter the fruit titratable acidity (Fig. 4e), in contrast to what reported for pepper^42^ and strawberry^27^, and positively affected the fruit firmness (Fig. 4b), which is an important qualitative trait for postharvest handling^38^. In fleshy fruits, such as tomato, characterized by thick and well-developed cuticles, the decrease of turgor may be indeed a primary cause of softening during ripening^43^ and iodine seemed to counteract this trend. We did not observe any influence of KIO_3_ on fruit color (Supplementary Fig. S3), thus suggesting a negligible role of iodine on the carotenoid metabolism^38^. Furthermore, the increased presence of iodine in the fruits, as an ordinary outcome of its administration^40^, represented an added value, considering the importance of this micronutrient in the human diet^17^.

The role of iodine to improve resilience to stress caused by mild or moderate salinity in tomato was then evaluated. In the growth chamber trial, iodine strongly mitigated the negative effects induced by the increasing salinity levels, and in its presence almost all the growth/production parameters were the same or even better than those measured in plants not treated with NaCl (Fig. 1). In the commercial-scale hydroponic system, the detrimental effects induced by a mild salinity stress^44,45,46^, when present, were again strongly alleviated by iodine (Figs. 2, 3). These results were consistent with a series of previous studies carried out, for example, in lettuce^3^ or strawberry^25^. In a recent study on tomato, three foliar applications of 100 μM KIO_3_ caused a 23% improvement in fruit yield in plants subjected to salinity stress (100 mM NaCl) during most of the growing cycle, without preventing the loss of plant biomass triggered by NaCl^47^. In accordance our data, the enhancement of fruit yield was associated with an increased number of fruits produced, also characterized by a higher FW *per* fruit^47^. Furthermore, if, as expected, fruit quality was positively influenced by salinity stress^45,46^, as attested by the increased fruit dry matter content (Fig. 3e), firmness and TSS (Fig. 4b,c), the concomitant treatment with iodine did not make worse the qualitative (Fig. 4) and nutritional (Supplementary Fig. S2) traits of tomato fruits.

Plant adaptation to saline conditions may include activation of different biochemical and physiological strategies aimed at restoring ion homeostasis^5^. In the present study, we did not measure the Cl^−^ and Na^+^ content in the tomato plants and fruits because the accumulation of these two ions is unlikely altered as a consequence of KIO_3_ treatments, at least when they are supplied in the range of concentrations used in the present experiments. Several studies performed on different crops reported the absence of a direct link between KIO_3_ treatments at low concentrations and plant ions/nutrients accumulation^48,50^, thus suggesting that the effects observed in our trials in KIO_3_-treated plants cannot be explained by iodine-induced mineral alternations.

On the contrary, we focused on the transcriptional analysis of a series of selected genes involved in other important mechanisms activated by plants in response to salt, to verify if their expressions were modulated by iodine or iodine + NaCl in different ways. We followed, in particular, the transcriptional response of genes involved in the activation of antioxidant systems in combination with ABA biosynthesis and signalling, because both these processes are of fundamental importance in the salt stress response^23^. High salinity in the soil can indeed reduce the ability of the plant to absorb water, leading to osmotic stress, and increased ABA production, and can also produce different oxidative stresses, counteracted by the activation of antioxidant systems^4^. Furthermore, previous bibliographic indications would suggest possible effects of iodine in these processes^3,15,16,47^. Thanks to this analysis, we observed that salinity stress was promptly perceived by the plants, which activated the stress marker genes since the first few days from the beginning of the salt administration (Fig. 5). In this early phase, however, the transcriptional activities controlling both the osmotic adjustments and the antioxidant response were less pronounced in plants exposed to NaCl and concomitantly treated with KIO_3_, suggesting that iodine attenuated the early responses to the stress. This finding was corroborated by the accumulation data of proline, which is one of the major endogenous osmolytes produced under salt stress^49^: iodine tended to reduce its accumulation in NaCl-treated leaves, sampled a few weeks after the onset of KIO_3_/NaCl treatments (Fig. 2e). Then, in a later stage of plant cultivation, this trend changed, as iodine tended to increase the expression of some of the same genes before activated by salinity and now not affected anymore by the only presence of NaCl (Fig. 6). It is possible that iodine, due to its low concentration, required some weeks of continuous administration to activate specific transcriptional responses against its presence, perceived in the long run as a new stress, overlapping with that earlier induced by salt and, in the end, resulting in a better plant adaptation to the same NaCl effects.

There is no previously published information to compare the transcriptomic effect of iodine in tomato plants exposed to salt stress with. However, at the biochemical level, several studies suggested a strong involvement of iodine in the antioxidant system of the plant, which in turn is strongly associated with plant resistance to abiotic stresses^3,15,16^. This could be in agreement with the induction at the gene expression level of the antioxidant response observed in the present study, when iodine was maintained, even if at low amounts, for a long period. It is then possible that short term responses of iodine led to an acute stress protection whereas long term responses allowed acclimatization to a chronic stress. The effects on the antioxidant response of the plants submitted to salinity and iodine at the same time thus appeared to be complex and worthy to be further examined.

## Conclusions

Our study reconfirmed that iodine, added as KIO_3_ at micromolar levels to the nutrient solution, can play fundamental roles in the primary and secondary metabolism of plants. In the absence of a concomitant stress, its administration during the whole life cycle of the plants resulted in a strong increase of tomato vegetative fitness and fruit yield, also significantly improving some fruit qualitative parameters. On the other hand, under a very common abiotic stress such as a mild salinity, addition of iodine with fertigation mitigated most of the negative effects induced by salt on plant growth and fruit yield, at different extents depending on the concentration of iodine or NaCl tested, without affecting the general positive effects exerted by salinity on fruit quality.

The physiological mechanisms allowing iodine to achieve such effects are at present not completely clear. However, the effectiveness even at the very low concentrations tested confirmed the direct involvement of iodine in plant nutrition, which could be different from (or additional to) its ability to increase the antioxidant activity, in accordance with our previous findings^15^. In both the cases, the concentration of iodine in the nutrient solution which is sufficient to elicit a benefit for crop production is comparable to recommended concentrations of other micronutrients of commercial horticultural crops.

## Methods

### Growth chamber experiment

#### Plant material and cultivation system

The tomato Micro-Tom cultivar was used. Plants were sown in 9 cm ø pots filled with a peat-based commercial substrate (Hawita-Flor®, Vechta, Germany) and, after vernalization, they were cultivated in a growth chamber under controlled light (80 µmol m^−2^ s^−1^ PAR), temperature (24 °C) and relative humidity (55%). Plants were fertigated three times per week with 10 ml of a basal nutrient solution prepared by dissolving in Milli-Q water appropriate amounts of ultrapure inorganic salts [KNO_3_, NH_4_NO_3_, Ca(NO_3_)_2_, Mg(NO_3_)_2_, KH_2_PO_4_, K_2_SO_4_], plus micronutrients. The concentration of macronutrients (mM) and micronutrients (µM) in the nutrient solution was the following: N–NO_3_ 14.0; N–NH_4_ 0.8; P 1.0; K 8.0; Ca 4.0; Mg 1.5; SO_4_ 2.4; Fe 45.0; B 20.0; Cu 1.0; Zn 5.0; Mn 10.0; Mo 1.0. At preparation, the electrical conductivity (EC) and pH of the nutrient solution ranged between 1.9–2.2 (dS·m^−1^) and 5.7–6.0 (adjusted with diluted H_2_SO_4_), respectively. Iodine concentration in the basal nutrient solution was below the detection limit of 8 nM, as determined by ICP-MS analysis. Two weeks after germination, plants were treated with iodine (0, 50 and 100 μM as KIO_3_) alone or in combination with NaCl (0, 25, 50 and 150 mM), by adding these compounds to the basal nutrient solution. The nutrient solutions with KIO_3_ and/or NaCl were provided to the plants until fruit collection (10 weeks, for a total number of 30 treatments). Twelve plants (biological replicates) were sown for each thesis.

#### Substrate electrical conductivity (EC)

At the end of the trial, the substrate EC was measured on soil–water extracts (1:2, by volume ratio). Briefly, two volume parts of demineralized water were slowly added to dried substrate samples and the suspensions were shaken for 20 min. A medium coarse filter paper was used for filtration, under vacuum, and the EC was determined in the extracts using a conductivity meter. Results confirmed the raised EC of the substrate in response to the different NaCl concentrations in the nutrient solution. On the contrary, iodine supplied alone or in combination with NaCl did not significantly affect the substrate EC (Supplementary Fig. S4).

#### Plant biomass and yield

At the end of the trial, plant vegetative parameters were characterized in terms of plant height, shoot FW and DW. For DW determinations tissues were dried at 70 °C in a ventilated oven until constant weight. Fruit yield was determined by collecting all the fruits and expressed on a DW basis.

### Greenhouse experiment

#### Plant material and cultivation system

The late-season cv. Cartesio F1 was used, which is a red, round shaped and cluster tomato, widely grown in temperate climates. Tomato plants were hydroponically cultivated inside a glasshouse located in central Italy (Pisa; N. 43.704282, W. 10.427033), from the middle of August to the beginning of December, under natural light conditions. During winter, the air temperature inside the greenhouse was maintained above 12 °C thanks to the use of electric hot-air heaters with fans, activated when necessary. Greenhouse climatic parameters were continuously monitored by means of a weather station located inside the glasshouse. The mean air temperature and relative humidity were 21 °C and 72.6%, respectively (T_min_ = 11.2 °C and daily T_max_ = 31.6 °C; RH_min_ = 49.3% and RH_max_ = 92.1%). Mean value of daily global radiation was 3.75 MJm^−2^ (GR_min_ = 0.9 MJm^−2^ and GR_max_ = 12.3 MJm^−2^). The hydroponic system was setup to grow each plant in an independent way on rockwool cubes (Grodan®—133 × 133 × 160 mm; 2 rock cubes/plant). Polystyrene holed plates were put on each cultivation bench to facilitate the drainage, thus avoiding any contamination between the run-offs and the plant root zone (plants treated with different nutrient solutions were placed on the same benches). An open hydroponic system was setup for the drip-irrigated substrate culture. During the first three weeks of cultivation, plants were fertigated with a basal nutrient solution, which was prepared by dissolving in tap water (iodine content averaged 0.03 µM, as determined by ICP-MS analysis) appropriate amounts of the ultrapure inorganic salts [Ca(NO_3_)_2_, KNO_3_, MgSO_4_, KH_2_PO_4_, K_2_SO_4,_ H_2_SO_4_; Fe-EDDHA, H_3_BO_3_, CuSO_4_, ZnSO_4_, MnSO_4_ and (NH_4_)_2_MoO_4_]. The concentration of macronutrients (mM) and micronutrients (µM) was the following: N–NO_3_ 12.8; P 1.0; K 8.4; Ca 5.0; Mg 1.5; SO_4_ 3.8; Fe 15.0; B 20.0; Cu 3.0; Zn 10.0; Mn 10.0; Mo 1.0. The EC and pH of the nutrient solution ranged between 2.3–2.5 dS/m^–1^ and 5.5–6.0 (adjusted with diluted H_2_SO_4_), respectively. After the first three weeks of cultivation, plants were divided in nine groups, each representing a different experimental condition. One group of plants (control) was fertigated with the basal nutrient solution throughout the life cycle. The other eight groups of plants were treated with iodine (0, 0.2, 10 µM KIO_3_) and NaCl (0, 30, 60 mM), supplied separately and/or in combination, by adding the salts to the basal fertigation solution. The EC of the solutions enriched with 30 and 60 mM NaCl was around 5.0 and 7.9 dS·m^–1^, respectively, whereas the pH was maintained between 5.5 and 6.0 by frequent adjustments with diluted H_2_SO_4_. Because of the low concentrations of iodine, the nutrient solution EC and pH were not affected by the presence of KIO_3_, irrespective of the concentration used. The frequency of plant watering was optimized to face plant requirements due to evapotranspiration: during the first phase of cultivation (the 1st month after the onset of iodine/NaCl treatments), the irrigation was supplied to plants for 1 min 3 times/day (8 A.M., 12 A.M. and 4 P.M.), whereas only two irrigation cycles were applied from the first middle of November until the end of the trial (1 min at 9 A.M. and 3 P.M.). Plants were allowed to grow till the production and ripening of fruits developed on the 5th truss, above which they were trimmed. For each experimental condition, 27 plant replicates, equally distributed on 9 different benches, were cultivated (Supplementary Figs. S5, S6). A periodical control of pests and pathogens was performed, by treating plants approximately every 15 days using different combinations of active ingredients (Supplementary Table S1).

### Determinations and measurements

#### Plant biomass and fruit yield

At harvest, plants were characterized in terms of vegetative parameters (plant height, shoot FW, DW and dry matter percentage calculated as DW/FW ·100; n = 13) and fruit yield, which was determined by counting and weighing all the produced fruits during the cultivation cycle, collected at the red ripening stage. Moreover, 1 fruit/truss was collected from each plant and used to determine the average FW, DW, and dry matter percentage of a single fruit. Data reported in graphs are the average values of fruits collected from the five different trusses per plant.

#### Proline content

Proline content was determined in leaf samples (terminal leaflets of comparable leaves collected three weeks after the beginning of the salt and iodate treatment. The same-age terminal leaflets (1 leaflet/plant; n = 27) were harvested and mixed to obtain a pool of material, which was analysed in triplicate for proline content, according to Carillo and Gibon^51^.

#### Fruit quality

The main qualitative traits, such as total soluble solids (TSS—expressed as °Brix), titratable acidity, firmness, peel colour, carbohydrate (glucose, fructose and sucrose), protein and iodine content, were characterized on fruits harvested in 2nd position of the 3rd truss cluster. All the fruits were collected at the same day, irrespectively of the time passed from the onset of anthesis. Fruit colour (one fruit/plant; n = 27; non-destructive method) and firmness (n = 12; destructive method) were determined at harvest on whole fresh fruits. The remaining collected material (27–12 = 15 fruits) was mixed, homogenized in a blender, divided into different sub-samples, and stored at −80 °C for the other qualitative determinations.

#### Content of total soluble solids, sugars, proteins and titratable acidity

The fruit homogenate was centrifuged twice for 10 min at 5000 rpm and the supernatant was used for the analysis. Titratable acidity was measured according to AOAC method 942.15^52^, whereas TSS were determined by a refractometer (RL3 type, PZO, Warszawa, Poland). The two parameters were expressed as citric acid % and °Brix, respectively. Glucose, fructose and sucrose content was quantified on fruit homogenate according to Guglielminetti et al.^53^, and expressed as µmol/g FW of tomatoes, whereas protein concentration was determined by bicinchoninic acid (BCA) assay (Thermo Scientific, Pierce BCA Protein Assay Kit), using bovine serum albumin standards. Analyses were performed in triplicate.

#### Fruit colour and firmness

Fruit colour was determined by using a Colorimeter (RGB 2, PCE Instruments, Southampton, United Kingdom) and expressed as HUE index. A digital penetrometer (catalogue number: 53205, TR Turoni, Forlì, Italy) was used to measure fruit firmness, expressed in Newton units (N).

#### Iodine content

Fruit iodine content was determined by Inductively Coupled Plasma-Mass Spectrometry (ICP-MS), as reported by Incrocci et al.^50^, and expressed as μg/kg FW of tomatoes.

#### RNA extraction, cDNA synthesis and gene expression analysis (RT-qPCR)

Transcriptomic analysis was performed on leaf samples collected at different time points during the plant cultivation (72 h and 8 weeks after the onset of NaCl and/or KIO_3_ treatments). Once sampled, leaves were immediately frozen in liquid nitrogen and stored at −80 °C until analysis. Total RNA was extracted using the Spectrum Plant Total RNA Kit (Sigma-Aldrich), according to the manufacturer's instructions. TURBO DNA-free kit (Thermo Fisher Scientific) was used to remove contaminant DNA, and the iScript DNA synthesis kit (Bio-Rad Laboratories, Hercules, CA, United States) was used for RNA reverse-transcription. Gene expression analysis was determined by quantitative PCR on an ABI Prism 7300 Sequence Detection System (Thermo Fisher Scientific) by processing 50 ng cDNA template with the iQ SYBR Green Supermix (Biorad Laboratories) and selected primer pairs on the following genes: *AREB1* (AY530758), a bZIP transcription factor^19^, *lptg2* (U81996.1), a lipid transfer protein^20^, *LEA* (*Solyc03g116390.2*), a late embryogenesis abundant protein^21^, *HAT9* (*Solyc02g063520.2*), a Homeobox-leucine zipper protein^21^, *CAT* (M93719.1), *SOD* (AY262025.1), *cytosolic Ascorbate Peroxidase, cAPX* (DQ099420.1), and *Glutathione Reductase, GR* (AW033378). *Elongation factor 1-alpha, EF1A* (X14449)^54^ and *Actin* (*Solyc03g078400.2.1*) were used as endogenous controls. Relative expression levels were calculated using the geometric averaging method (GeNorm)^55^. The list of primers used, and their sequences are reported in Supplementary Table S2. Three biological replicates were analyzed, each consisting in a pool of leaves sampled from different plants.

### Statistical analysis

Data were analysed by one-way ANOVA coupled with the LSD post hoc test, when they followed a normal distribution and there was homogeneity of variances. When one of these two prerequisites was violated, a Kruskal–Wallis test for non-parametric statistic was performed and the significance letters were graphically assigned using a box-and-whisker plot with a median notch. Significant differences between the means/medians (P < 0.05) are indicated by different letters in each graph.

### Ethical approval

Experimental research on plants including collection of plant material, complied with institutional, national, and international guidelines.

## Supplementary Information

Supplementary Information 1.

Supplementary Information 2.

## Data Availability

All data generated or analysed during this study are included in this published article (and its Supplementary Information files).
