# Supplementary material for: Improvement in fruit yield and tolerance to salinity of tomato plants fertigated with micronutrient amounts of iodine
Source: Sci Rep. 2022 Aug 29;12:14655. doi: 10.1038/s41598-022-18301-w (PMC9424290; doi:10.1038/s41598-022-18301-w)
Supplement: Supplementary file 1 — Supplementary Information 1. [file 41598_2022_18301_MOESM1_ESM.pdf]

Improvement in fruit yield and tolerance to salinity of tomato plants fertigated with micronutrient amounts of iodine

Claudia Kiferle[1], Silvia Gonzali[1], Sara Beltrami[1], Marco Martinelli[1], Katja Hora[2], Harmen Tjalling Holwerda[2], Pierdomenico Perata[1]

[1]PlantLab, Center of Plant Sciences, Scuola Superiore Sant'Anna, Pisa, Italy

[2]SQM International N.V., 2030 Antwerpen, Belgium

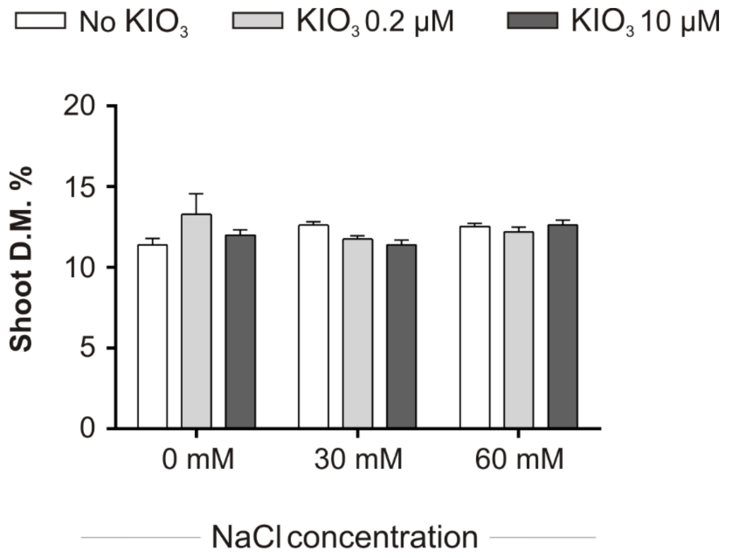

Supplementary Figure S1. Greenhouse experiment: impact of iodine on shoot dry matter percentage (D.M. %). Each bar is the mean (±SE) of 13 replicates, each consisting of one individual plant. Data did not follow a Normal distribution, therefore, a Kruskal-Wallis test was performed. Since the P-value is greater than or equal to 0.05, there is not a statistically significant difference amongst the medians at the 95.0% confidence level.

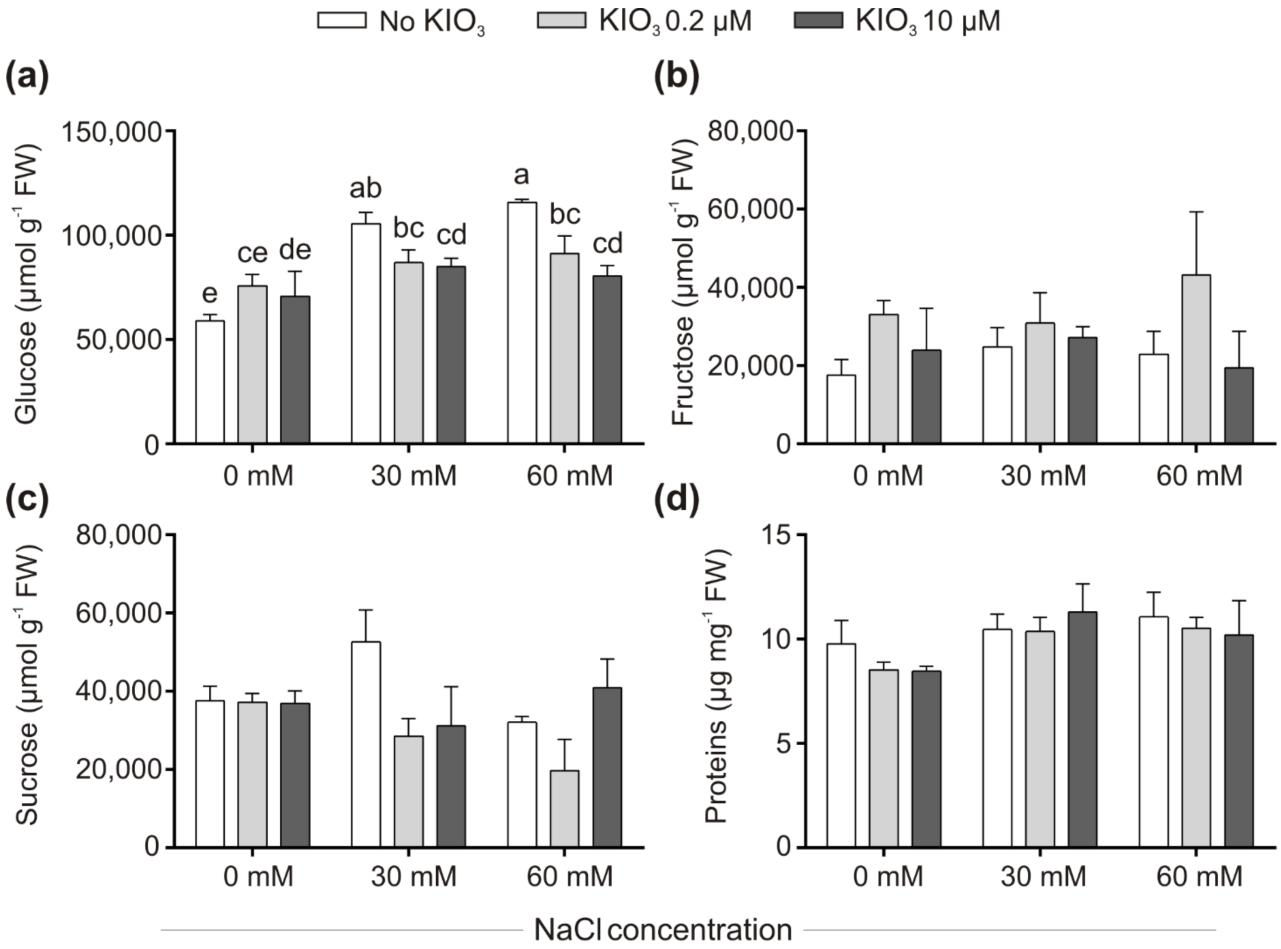

Supplementary Figure S2. Impact of iodine on fruit glucose (a), fructose (b) and sucrose (c) content in the absence/presence of salt stress. Each bar is the mean (±SE) of 3 replicates, each consisting of a sub-sample produced through homogenization of 15 fruits. When data followed a Normal distribution and there was homogeneity of variances, they were subjected to one-way ANOVA and values indicated by different letters significantly differ from each other (LSD post hoc test, $P \leq 0.05$). When one of this two prerequisites was violated, a Kruskal-Wallis test was performed and significant differences within medians were determined by Box-and-Whisker Plot (median notch option, $P \leq 0.05$) and indicated by different letters.

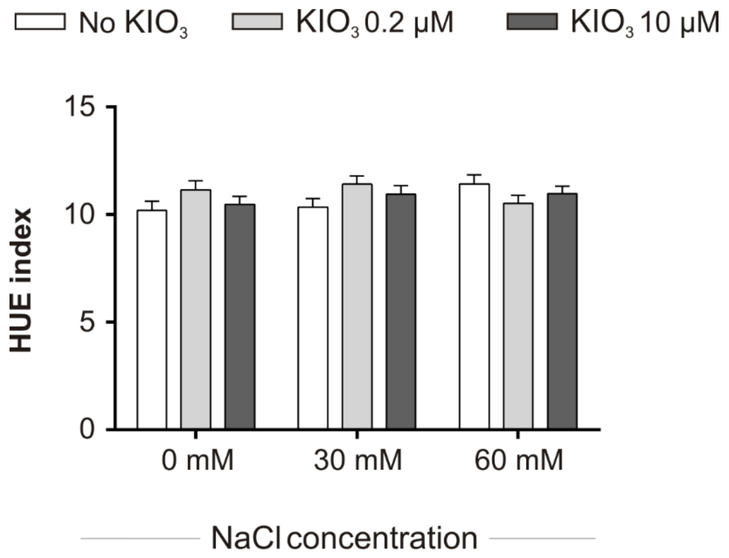

Supplementary Figure S3. Impact of iodine on fruit peel colour, expressed as HUE index, in the absence/presence of salt stress. All the produced fruits harvested in 2nd position of the 3rd truss cluster were analyzed (n=27). Data did not follow a Normal distribution, therefore, a Kruskal-Wallis test was performed. Since the P-value is greater than or equal to 0.05, there is not a statistically significant difference amongst the medians at the 95.0% confidence level. Error bars (±SE) are shown in graphs.

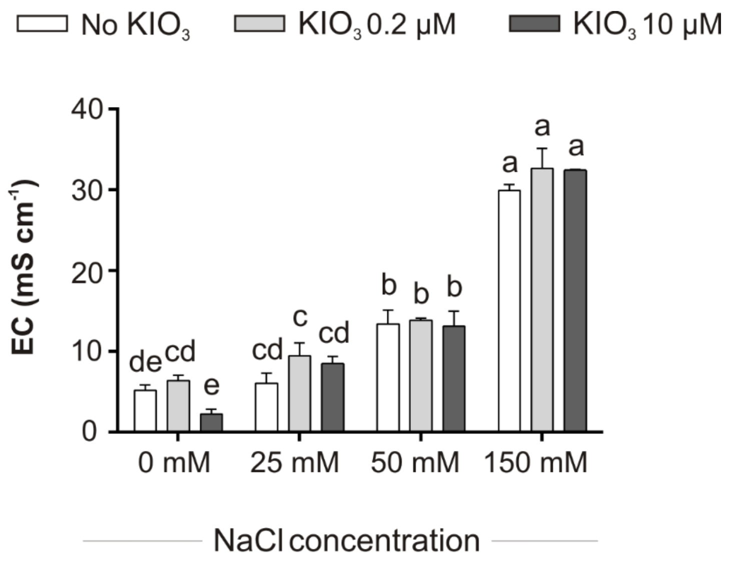

Supplementary Figure S4. Electrical conductivity (EC) measured in the substrate extracts at the end of the trial. Each bar is the mean (±SE) of 3 replicates. Data did not follow a Normal distribution, therefore, a Kruskal-Wallis test was performed. Different letters indicated significant differences amongst the medians at the 95.0% confidence level.

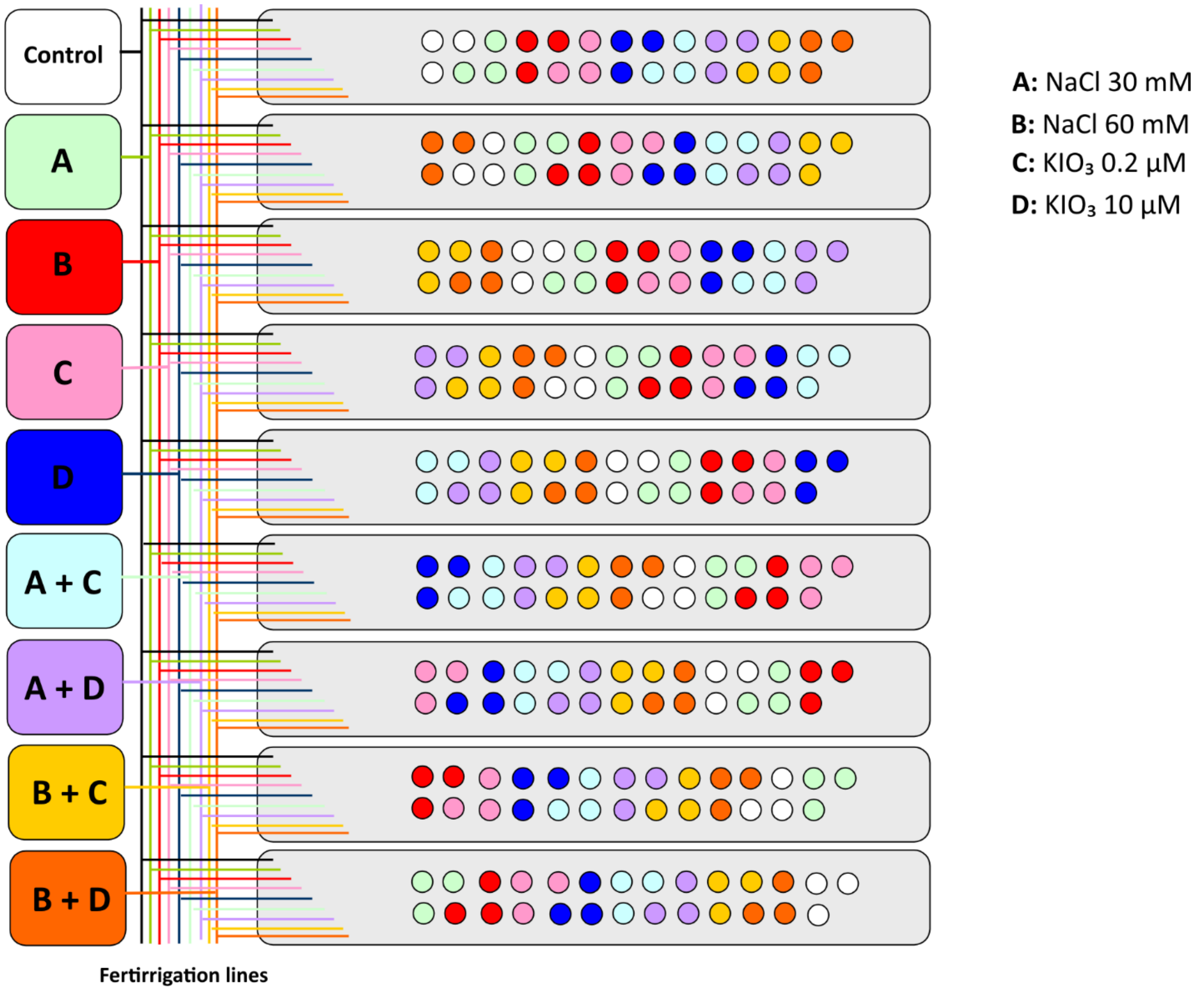

Supplementary Figure S5. Experimental design of the greenhouse trial. Coloured circles represent single plants, fertigated with the different nutrient solutions indicated on the left. Totally, 243 plants were cultivated. 27 biological replicates, each represented by a single plant, were grown for each experimental condition. Three plants for each of the nine experimental conditions were cultivated on each bench.

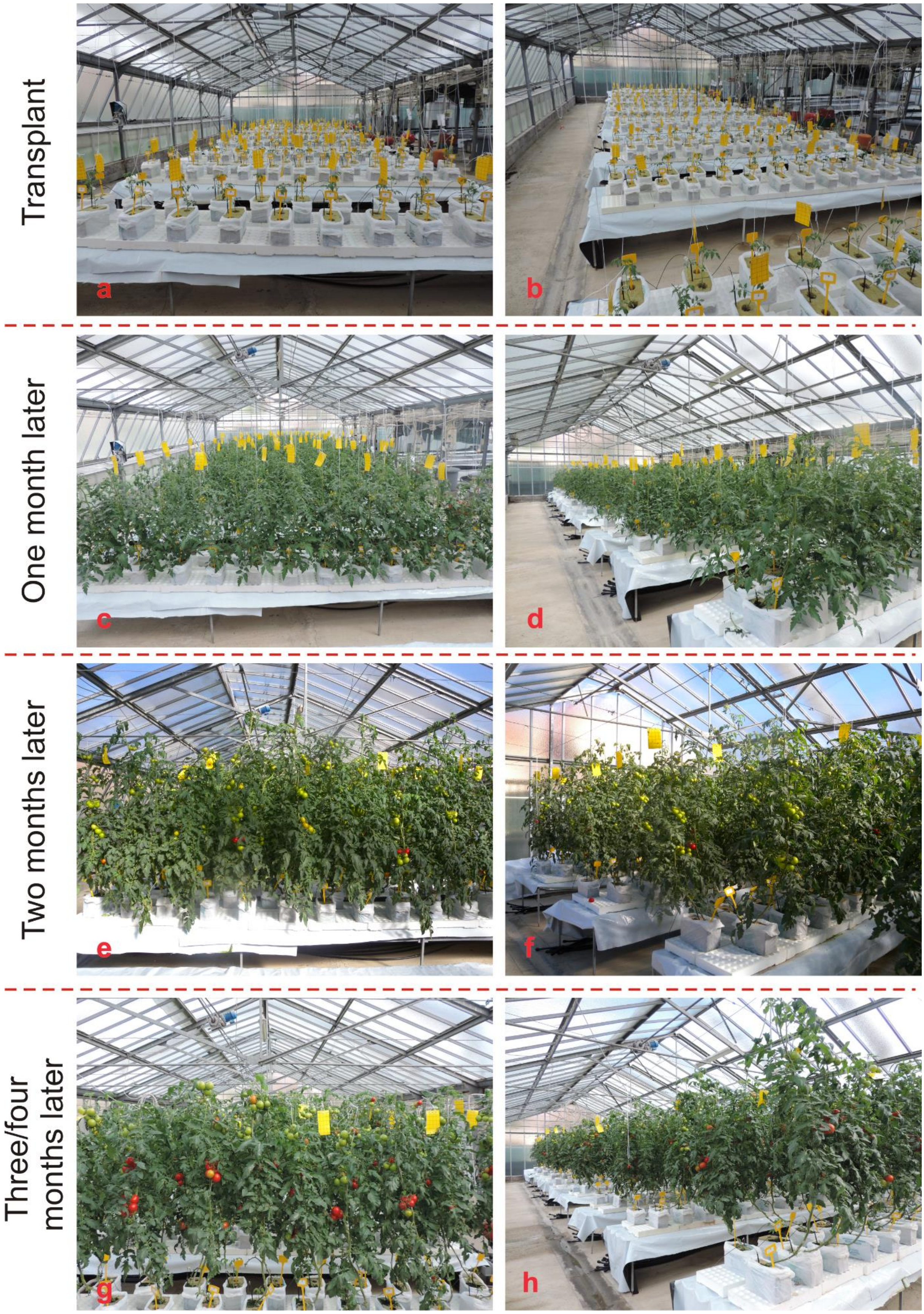

Supplementary Figure S6. Different steps of tomato hydroponic cultivation in the greenhouse. Plants at the transplant (a-b) and approximately after one (c-d), two (e-f) and three-four months of cultivation (g-h) are shown.

Supplementary Table S1. List of pesticides used in the trial.

Commercial name	Description	Main targets
Ridomil Gold®	systemic fungicide	oomycete "fungi"
Ortiva®	systemic and contact fungicide	Botrytis and white rust
Confidor®	systemic insecticide	aphids, thrips, mealybugs
Decis®	contact and ingestion	aphids, caterpillars, whitefly
Vertimec®	translaminar acaricide/insecticide	acarids, thrips, psylla, leafminers
Calipso®	systemic and contact insecticide	aphids, psylla, whitefly
Safran®	translaminar acaricide/insecticide	acarids, leafminers, psylla

Supplementary Table S2. Primers used for RT-qPCR gene expression analysis.

Gene	Encoding protein	Gene ID	Primer pairs
AREB1	ABA-Responsive Element Binding Protein 1	AB017160	F: CAGGTTTAATGGCTGGTAGTATCCC<br>R: GCTGTGATTGTTGGTTCTGTTGC
ltpg2	Non-specific lipid transfer protein GPI-anchored 2	U81996.1	F: TACTGGACCGTTGAGCA<br>R: GGTGTTGTGGTGGTGTTA
LEA	Late embryogenesis abundant protein	*Solyc03g116390.2*	F: CAATGTCTCACGAGCAGAGC<br>R: TGCTTTCCAGTGTTTGACCA
HAT9	Homeobox-leucine zipper protein 22	*Solyc02g063520.2*	F: CTTGGCAAGGAAGCTTAACG<br>R: CTCGCAATCCACCTCTGTTT
SOD	Superoxide dismutase	AY262025.1	F: GGCTTGCATACAAACCTGAA*<br>R: CTGACTGCTTCCCATGACAC*
CAT1	Catalase 1	M93719.1 F	F: GTCGATTGGTGTTGAACAGG*<br>R: AGGACGACAAGGATCAAACC*
cAPX1	cytosolic Ascorbate Peroxidase 1	DQ099420	F: GACTCTTGGAGCCCATTAGG*<br>R: AGGGTGAAAGGGAACATCAG*
GR	glutathione reductase	AW033378	F: TTGGTGGAACGTGTGTTCTT*<br>R: TCTCATTCACTTCCCATCCA*
EF1A	Elongation factor	X14449	F: TGCTTGCTTTCACCCTTGGT<br>R: CGATTTCATCATACCTAGCCTTGGA
Actin	Actin	*Solyc03g078400.2.1*	F: TGGGATGATATGGAGAAGATATGG*<br>R: CTCAGTCAGGAGAACAGGGT*

F: forward primer; R: reverse primer.
*Sequences are from Liu et al.[20].
